# The incidence and outcome of septic shock patients in the absence of early-goal directed therapy

**DOI:** 10.1186/cc4918

**Published:** 2006-05-16

**Authors:** Benjamin CH Ho, Rinaldo Bellomo, Forbes McGain, Daryl Jones, Toshio Naka, Li Wan, George Braitberg

**Affiliations:** 1 Department of Intensive Care, Department of Medicine, University of Melbourne, Melbourne, Australia; 2 Department of Emergency Medicine, Austin Hospital, Melbourne, Australia

## Abstract

**Introduction:**

The purpose of the present study was to measure the incidence and outcome of septic patients presenting at the emergency department (ED) with criteria for early goal-directed therapy (EGDT).

**Method:**

This hospital-based, retrospective, observational study using prospectively collected electronic databases was based in a teaching hospital in Melbourne, Australia. We conducted outcome-blinded electronic screening of patients with infection admitted via the ED from 1 January 2000 to 30 June 2003. We obtained data on demographics, laboratory and clinical features on admission. We used paper records to confirm electronic identification of candidates for EGDT and to study their treatment. We followed up all patients until hospital discharge or death.

**Results:**

Of 4,784 ED patients with an infectious disease diagnosis, only 50 fulfilled published clinical inclusion criteria for EGDT (EGDT candidates). Of these patients, 37 (74%) survived their hospital admission, two (4%) died in the ED, eight (16%) died in the intensive care unit and three (6%) died in the ward. After review of all ward cardiac arrests and non-NFR ('not for resuscitation') ward deaths, we identified a further two potential candidates for EGDT for an overall mortality of 28.8% (15 out of 52 patients). Analysis of treatment showed that twice as many (70%) of the EGDT candidates received vasopressor therapy in the ED, and their initial mean central venous pressure (10.8 mmHg) was almost twice that in patients from the EGDT study conducted by Rivers and coworkers.

**Conclusion:**

In an Australian teaching hospital candidates for EGDT were uncommon and, in the absence of an EGDT protocol, their mortality was lower than that reported with EGDT.

## Introduction

Patients with the clinical diagnosis of an infectious disease are frequently admitted to hospital from the emergency department (ED). Some of these patients have severe sepsis or septic shock and require admission to the intensive care unit (ICU). Rivers and coworkers [1] recently reported that resuscitation with an approach described as early goal-directed therapy (EGDT) reduced mortality in such patients from 46.5% to 30.5%. Accordingly, EGDT, if widely applicable and generalizable, potentially could save thousands of lives worldwide. In response to this evidence, many centers have considered the introduction of EGDT to the care for their ED patients presenting with both severe sepsis and appropriate criteria for EGDT.

However, no information exists outside the single center in which the EGDT study was conducted. In particular, there is no information on how frequently such patients attend the ED in different health care systems, institutions, or geographical settings, and on their mortality outside the single institution in which the EGDT trial was conducted. Such information would be of value in improving our understanding the external validity of the EGDT approach, assessing the possible workload for a sepsis team, clarifying the 'therapeutic opportunity' associated with EGDT and helping other investigators to power multicenter randomized controlled trials of EGDT. The US National Institutes of Health recently funded one such study (ProCESS), which will conduct preliminary feasibility work prior to randomization.

In our institution, EGDT has not yet been implemented but was considered as an important quality improvement measure. Accordingly, we sought to obtain institution-specific baseline information relevant to EGDT and studied the incidence and outcome of septic patients admitted to hospital through our ED who fulfilled criteria for EGDT. Here we report our findings.

## Materials and methods

### Study population

This study was approved the Human Research Ethics Committee of the Austin Hospital. The need for informed consent was waived because the study required no intervention and no breach of privacy or anonymity. The Austin Hospital is an urban, academic, teaching, tertiary care hospital with 800 beds that is based in the city of Melbourne, Australia. The ED of the Austin Hospital has approximately 40,000 patient visits per year, with approximately 12,000 of these resulting in hospital admission.

We included all adult patients (age >18 years) who were admitted to the Austin Hospital from the ED with an admission ICD-10 (International Classification of Disease version 10) diagnostic code of an infectious condition (Table 1). We studied a period of 3.5 years (from 1 January 2000 through 30 June 2003), which preceded and overlapped with the timing of the EGDT study conducted by Rivers and coworkers [1]. We called this group the 'primary cohort'. The selection process was blinded to outcome.

From this primary cohort we then identified patients with severe sepsis or septic shock by selecting all patients who died in the ED, required care in the ICU (including all patients who died in the ICU), required care in the high dependency unit (HDU; including all patients who died in the HDU), died in the general ward from cardiac arrest, and died in the general wards without a 'not for resuscitation' (NFR) order. We called this the 'secondary cohort'.

For this secondary cohort we reviewed all charts and electronic ED records. This allowed us to identify patients who met the following EGDT criteria while in the ED (as described by Rivers and coworkers [1]): two of four criteria for the systemic inflammatory response syndrome [2] and a systolic blood pressure no higher than 90 mmHg (after a crystalloid fluid challenge of 20–30 ml/kg body weight over 20–30 minutes). We recorded these patients as meeting the 'clinical criteria' for EGDT. However, in the EGDT study [1] patients could also be randomized because of a lactate concentration above 4 mmol/l (biochemical criterion for EGDT). Accordingly, by linking with the central laboratory electronic database, we also identified all patients from the primary cohort with a lactate concentration above 4.0 mmol/l. In those patients in whom the lactate concentration was not measured, we used a base excess (BE) of -4 mEq/l or worse or a bicarbonate below 20 mmol/l as surrogates for a lactate of 4 mmol/l. To ensure that no patients were missed who might have had a lactate concentration above 4.0 mmol/l, we conducted a further 'tertiary analysis' using a BE of -3 mEq/l or worse as the cut off point. Finally, we conducted a fourth-round analysis using a BE of -2 mEq/l or worse as the selection criterion.

**Figure 1:**
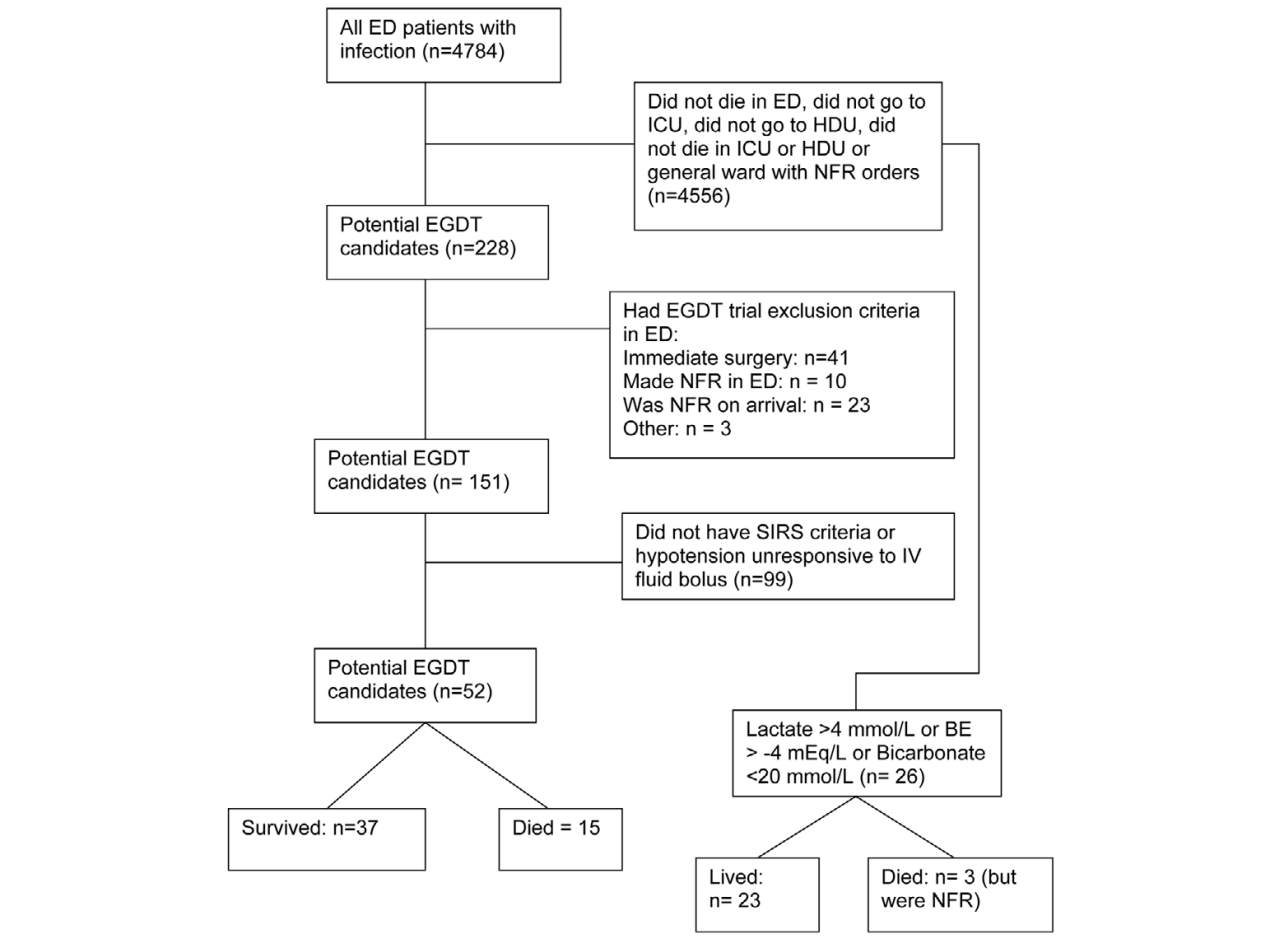
Study profile. BE, base excess; ED, emergency department; EGDT, early goal-directed therapy; HDU, high dependency unit; ICU, intensive care unit; IV, intravenous; NFR, not for resuscitation; SIRS, systemic inflammatory response syndrome.

All patients included at each step as potential candidates for EGDT were defined as meeting the 'laboratory criteria' for EGDT and added to the secondary cohort. Patients were, of course, excluded from our study if they fulfilled one or more of the exclusion criteria described in the EGDT trial [1]. This selection process is summarized in the study profile given in Figure 1.

For all patients in the secondary cohort medical records were retrieved to obtain data on history, vital signs, laboratory results, treatment and details of outcome using the central laboratory database, the ICU patient information database, the hospital admissions and discharges database (which records all deaths) and the hospital cardiac arrests database. The severity of illness of the study patients was calculated using the Acute Physiology and Chronic Health Evaluation (APACHE) II score [3] and the Simplified Acute Physiology Score II [4].

Data were compiled and analyzed using Microsoft Excel 2002. Additional statistical analysis was performed using Statview, version 4.57 (Abacus Concepts, Inc., Berkeley, CA, USA). Results for descriptive statistical analysis are expressed as means with standard deviation. *P *< 0.05 was considered statistically significant.

## Results

### Patients

Over the study period, 4,784 patients were admitted via the ED with a diagnosis of an infectious disease or probable infectious disease. From this cohort, 4,556 patients were excluded from further analysis because they did not die or go on to the ICU or the HDU. This left 228 candidates for EGDT (Figure 1). Of these, 23 had pre-ED NFR orders and were excluded, in accordance with the exclusion criteria employed by the EGDT trial [1]. There were a further 41 septic patients who required immediate (within 2 hours) surgery. Because immediate surgery was an exclusion criterion of the EGDT trial, these patients were also excluded. Three patients met other exclusion criteria, as described in the EGDT study. Another 10 patients had NFR orders assigned to them in the ED after discussion with the family and primary unit. These patients were also excluded from analysis, as described in the EGDT trial. Two patients remained who died in the ED without NFR orders and met the study criteria. They were included in the outcome analysis. A further 99 patients had sepsis, were admitted to ICU or HDU but never met the clinical EGDT study criteria for severe sepsis or septic shock. This left only 50 patients with sepsis who met the clinical study criteria and none of the exclusion criteria. Importantly, no patients with severe sepsis were directly admitted to the ICU from another hospital during this time and no patients with severe sepsis were transferred from the ED of our hospital to another hospital during the study period. Thus, a total of 52 (50 admitted to the ICU/HDU + 2 who died in the ED without NFR orders) patients fulfilled the clinical study criteria for severe sepsis or septic shock used in the EGDT study [1] and none of its exclusion criteria.

However, during the study period, there were also 104 cardiac arrests and/or non-NFR deaths in the general wards. Of these, only two patients had been admitted via the ED with infection. Both were older than 80 years and had multiple co-morbidities (ischaemic heart disease, chronic obstructive airways disease, asbestosis diabetes, chronic renal failure). They did not appear to fulfill either clinical or BE or bicarbonate criteria for EGDT while in ED.

### Treatment and outcomes

The average time spent in the ED by the EGDT candidates before hospital admission was 8.5 ± 5.3 hours (median 7.9 hours, range 2 to 26.9 hours). The mean total hospital length of stay from presentation at the ED was 13.3 ± 12.2 days (median 9.6 days, range 0.1 to 64.8 days).

Three of these patients were initially admitted to the general ward before being transferred to the ICU. Of the 17 patients admitted to the HDU, three were subsequently transferred to the ICU after being in the HDU for 6 to 23 hours. The mean HDU LOS for these 17 patients was 2.4 ± 3.8 days (median 1.0 days, range 0.2 to 15.8 days). The number of patients admitted directly to the ICU was 28. The mean ICU length of stay for these 34 patients (including the six patients who were transferred into it) was 3.2 ± 3.4 days (median 2.0 days, range 0.3 to 14.6 days).

The clinical and laboratory findings during the first 6 hours in the ED are summarized in Table 2. Four patients had temperatures below 36.0°C and 31 had temperatures greater than or equal to 38.0°C. Of the 49 patients in whom blood culture was performed, 17 had positive blood cultures. Of the 41 patients in whom urine culture was performed, seven were positive; and of the 22 patients in whom sputum or tracheal aspirate culture was performed, five were positive. Table 3 summarizes their primary sources of infection.

While in the ED, among the 50 patients admitted to hospital, 39 (78%) patients had an intra-arterial catheter inserted. For the 36 (72%) patients who had a central venous catheter inserted in the ED, the mean initial central venous pressure was 10.8 ± 4.7 mmHg (median 11 mmHg, range 2–20 mmHg). Only one patient (2%) received a blood transfusion.

Antibiotics were started within 6 hours in 45 (90%) patients. Vasopressor agents were started in the ED in 35 (70%) patients. Mechanical ventilation was commenced in the ED in 12 (24%) patients. Another three patients subsequently required mechanical ventilation in the ICU.

Of these 52 study patients, 37 (71.2%) survived hospital admission, two (3.8%) died in the ED, eight (15.3%) died in the ICU and six (11.5%) died in the ward.

Using the lactate criterion, an additional single patient was found to have been a candidate for EGDT (lactate of 4.8 mmol/l) who had not been identified using the clinical criteria. This patient was a 20-year-old female with pyrexia of unknown cause. She was admitted to a general ward and survived to hospital discharge.

Using a BE of -4 mEq/l or worse or a bicarbonate below 20 mmol/l as markers for an elevated lactate concentration when lactate was not measured, 26 patients were found who might also have been candidates for EGDT. Of these 26 patients, 23 survived to hospital discharge. The three who died had NFR orders. Using a BE of -3 mEq/l or worse, a further four patients were identified who might have been candidates for EGDT. Of these, three survived to hospital discharge. The one who died had a NFR order. Using a BE of -2 mEq/l or worse, an additional eight patients were identified. Of these, six survived to hospital discharge. The two who died had NFR orders.

## Discussion

We conducted a study of patients presenting to the ED of a teaching hospital in Melbourne, Australia with severe sepsis or septic shock and who met clinical criteria for EGDT [1]. We measured the incidence of candidacy for EGDT and mortality rates in such patients. We found that approximately 1.0% of patients with a diagnosis of infection were candidates for EGDT. In the absence of EGDT, hospital mortality in these patients was 30.2%. We further made comprehensive attempts to identify any other patients who might have had an elevated lactate in isolation as the sole criterion for EGDT. After their inclusion, the incidence of the syndrome (reflected by candidacy for EGDT) was still only 1.6% and its non-NFR mortality lower. These findings are relevant to other institutions considering the application of EGDT and to the powering of future randomized controlled trials.

It must be borne in mind that this (like the EGDT trial [1]) is a single centre study. Thus, our observations might not apply to other centres. However, this is precisely the point and major finding of our investigation, namely that in different health care systems, geographical settings and institutions the syndrome described in the EGDT trial may be relatively uncommon and carry a different mortality. However, we note that our findings are concordant with those reported by Shapiro and coworkers [5]. Those American investigators identified ED patients at risk for infection, as indicated by blood culture order. Of the 2,070 patients so identified, only 52 (2.5%) had septic shock (defined as severe sepsis plus persistent hypotension after an initial fluid challenge of 20–30 ml/kg), with a 28-day hospital mortality rate of 26.9%. This mortality rate is similar to that in our study. Using two electronic reference libraries and broad search strategies, we could not find any other studies of the outcome of ED patients with severe sepsis or septic shock.

Our study is retrospective, with all the limitations inherent to such studies. However, we used a specific and reproducible strategy to identify patients for inclusion, making it possible for other investigators to confirm or refute our findings elsewhere. Furthermore, the data used were electronically and prospectively collected and stored in various hospital databases, and were not amenable to bias or manipulation. Some candidates for EGDT might have been admitted to the general ward and missed by our identification process. However, because of our hospital's policy, all patients requiring vasopressor therapy or who remain hypotensive despite fluid resuscitation are admitted to the ICU or HDU in our institution. If any such patients were ever admitted to the general wards, it would be for palliative care only. This would have excluded them from allocation to EGDT. Nonetheless, we studied all deaths to ensure that no possible EGDT candidates who died would be missed. Only two such patients were identified who had died in the ward and were not NFR. These patients did not fulfill the clinical EGDT trial criteria and their base deficit was not greater than -3 mEq/l. Nonetheless, according to study protocol, these two patients who died were added to the overall cohort.

We might have missed patients who did not fulfill clinical criteria for EGDT but who would have been randomized to EGDT on the basis of an isolated lactate concentration above 4 mmol/l. (We note that the EGDT trial did not report how many such patients were randomized on the basis of an elevated lactate only.) However, only one patient had a lactate above 4 mmol/l in isolation. That patient survived. Because lactate was not routinely measured in all septic patients admitted to hospital, we used BE and bicarbonate as surrogates to identify patients who might have had an isolated yet unmeasured high lactate level. We thus identified several such patients and included them in our analysis. Their inclusion further lowered the mortality rate and only slightly increased the number of possible candidates for EGDT. We note that our protocol was biased toward inflating mortality by identifying all deaths among the initial cohort and potentially missing patients who fulfilled the clinical or biochemical criteria for EGDT but went on to be treated in the ward and did not die.

Some patients might have had severe sepsis or septic shock but were not identified by the ED ICD coding, medical and nursing notes, and hourly observations. This is unlikely. However, even if this were true, these patients would not have been candidates for EGDT, because such treatment requires recognition of sepsis as the cause of the patient's illness. We might have incorrectly classified patients as having severe sepsis or septic shock when in fact they were less ill. This is essentially impossible because the search criteria were prospectively determined, numerical and objective in nature, and could only be verified with independently recorded observations or laboratory findings.

The low incidence of the syndrome targeted by EGDT in our hospital may reflect a particular health care system, geographical setting, or institution. However, this is the very point we wish to make – each hospital must assess its characteristics with regard to this syndrome and its outcome. We note, however, that the incidence found in our hospital is concordant with that reported by Shapiro and coworkers [5]. We also note that our hospital is typical of a teaching hospital in any large Australian city. However, the Australian health care system is quite different from the American system and offers free access to medical care to all patients, irrespective of health insurance status. Some patients with sepsis might present much later in the US system because concern about lack of health insurance and associated cost of care.

The low mortality in our cohort might reflect a group that was less critically ill. This once again supports our point about the need for each institution to assess its own population. Nonetheless, the mean APACHE II score was 19.8 versus 20.4 in the EGDT study; the percentages of patients with severe sepsis and septic shock in our study were 44.0% and 56.0%, respectively, compared with 48.7% and 51.3% in the control group of the EGDT study. Furthermore, our mortality rate is concordant with the findings reported by Shapiro and coworkers [5] and those of the recent PROWESS trial [6], which reported 30.8% mortality in septic patients randomly assigned to placebo (pre-randomization APACHE II score 25). The mortality reported in the control arm of the EGDT trial, however, is discordant with these findings.

Many of our patients were assigned to palliative care only. Some of these patients might have been considered for active therapy elsewhere and received active intervention in the EGDT study. Such patients might have died more frequently and thus led to a high mortality. However, if this were the case then this difference would provide further proof of the need to assess more broadly the generalizability of the EGDT approach.

Finally, the reported advantages of EGDT might not reflect the style of fluid and haemodynamic management *per se *[7-9] but rather the general consequences of early involvement of expert physicians in the management of severe sepsis and septic shock. Early experienced team intervention, rather than specific interventions targeted at high superior vena cava oxygen saturation, may be the variable within the EGDT intervention bundle that improves survival. If this is so, then in the model of care operative at our institution (formal training in emergency medicine, senior emergency medicine staff rapidly involved in patient care 24 hours/day, early co-management and 'closed' ICU system) such advantages might already be realized. The intervention rates of central line insertion in 78% of patients, arterial cannulation in 72% of patients, and antibiotic administration in the first 6 hours in 90% of cases all support this view. More importantly, the initial mean central venous pressure CVP of above 10 mmHg in our patients (compared with between 5 and 6 mmHg in the EGDT study [1]) and a 70% rate of vasopressor drug administration (compared with approximately 30% in the EGDT study) suggest that more aggressive and prompt resuscitation was already taking place routinely in our hospital even before the publication of the EGDT protocol.

## Conclusion

In a teaching hospital in Australia, the number of candidates for EGDT was low, mortality lower than that reported with EGDT and treatment already potentially more aggressive and prompt. These observations provide useful information for the powering of planned multicentre randomized controlled trials of EGDT. They now require confirmation in other geographical and health care settings.

## Key messages

• 	Of more than 4,700 patients with an infectious disease diagnosis, little more than 1% fulfilled criteria for EGDT.

• 	Among patients who were candidates for EGDT, 70% were treated with vasopressor drugs.

• 	For patients who were candidates for EGDT, the mortality rate was 28.8%.

• 	The incidence of candidacy for EGDT may be lower than widely believed and outcome may be better than estimated, even in the absence of EGDT; this information is important in that it may help to design planned multicentre trials of EGDT.

## Abbreviations

APACHE = Acute Physiology and Chronic Health Evaluation; BE = base excess; ED = emergency department; EGDT = early goal-directed therapy; HDU = high dependency unit; ICD-10 = International Classification of Disease version 10; NFR = not for resuscitation.

## Competing interests

The authors declare that they have no competing interests.

## Authors' contributions

BH, FM, DJ, TN and LW conducted data analysis and assisted with manuscript development. RB developed the study design and assisted with data analysis and manuscript development. GB participated in the data interpretation and manuscript development. All authors read and approved the final manuscript.

## Figures and Tables

**Table 1:** Diagnoses of infections by ICD-10 codes for patients admitted from the emergency department

Code	Diagnosis	Number
A09A	Enteritis, presumed infectious. Excludes noninfectious enteritis K52.9	21
A09B	Gastroenteritis, presumed infectious. Excludes noninfectious gastroenteritis K52.9	98
A09C	Diarrhoea. Excludes diarrhoea noninfectious K52.9	32
A41.8	Septicaemia	278
A41.9	Septic shock	45
A49.9	Bacteraemia (excludes that with sepsis, see Septicaemia)	16
A86	Encephalitis, viral (includes viral meningoencephalitis/viral encephalomyelitis)	7
J02.0	Pharyngitis, acute streptococcal (includes sore throat)	9
J03.9	Tonsillitis, acute	36
J18.0	Bronchopneumonia	434
J18.1	Pneumonia, lobar	928
J20.9	Bronchitis, acute	24
J22A	Acute lower respiratory tract infection	500
J22B	Lower respiratory tract infection, chest	82
J69.0	Bronchopneumonia, aspiration	119
K04.7	Abscess, dental	16
K22.3	Perforation of oesophagus	2
K27.1	Peptic ulcer, acute, with perforation	14
K61.0	Abscess, perianal or anal	67
K81.9	Cholecystitis	165
L03.9	Cellulitis, skin, any site. Excludes cellulitis of finger or toe L03.01/L03.02	541
M00.99	Arthritis, infective	36
N12	Pyelonephritis	124
N30.9	Cystitis	1
N39.0A	Bacteriuria/urinary tract infection (UTI)	230
N39.0B	Urinary sepsis	178
N39.0C	Urinary tract infection	226
N45.9C	Epididymitis/orchitis/epidymo-orchitis	33
R09.1	Pleurisy	23
R10.0	Severe abdominal pain/acute abdomen	90
R19.8	Perforated viscous/ruptured viscous	31
R50.9	Fever/pyrexia of unknown origin (PUO)	378
	Total	4,784

ICD-10, International Classification of Disease version 10.

**Table 2:** Clinical and laboratory findings in the emergency room

Parameter	Value
Highest heart rate	119 ± 25 beats/minute
Highest respiratory rate	31.7 ± 8.9 breaths/minute
Mean APACHE II score	19.8 ± 7.8
Mean SAPS II score	40.2 ± 16.1
Mean white cell count	13.2 ± 10.1 × 10^9^/l
Mean haemoglobin	12.4 ± 2.2 g/dl (lowest value: 7.8 g/dl)
Mean platelet count	217 ± 218 × 10^9^/l
Mean [urea]	13.6 ± 7.6 mmol/l
Mean [creatinine]	222 ± 218 μmol/l

APACHE, Acute Physiology and Chronic Health Evaluation; SAPS, Simplified Acute Physiology Score.

**Table 3:** Primary source of infection in patients admitted to the ICU/HDU with clinical criteria for EGDT

Diagnosis	Number of patients
Lower respiratory tract infection	19
Unknown source	10
Urinary tract infection	9
Abdominal infection	4
Central nervous system infection	3
Skin/muscle infection	3
Vascular line infection	1
Dental infection	1

EGDT, early goal-directed therapy; HDU, high dependency unit; ICU, intensive care unit.
